# P-1152. Cefepime, Meropenem, and Piperacillin Therapeutic Drug Monitoring in Neonatal and Pediatric Patients

**DOI:** 10.1093/ofid/ofae631.1338

**Published:** 2025-01-29

**Authors:** Laura Dinnes, Elizabeth H Ristagno, Jane E Brumbaugh, Raymond Stetson, Emily Ruth Levy, Grace Lee, Christina G Rivera, Brandi Smith, Gregory Thurber, Taylor Gullickson, Erin F Barreto

**Affiliations:** Mayo Clinic, Rochester, Minnesota; Mayo Clinic, Rochester, Minnesota; Mayo Clinic College of Medicine and Science, Rochester, Minnesota; Mayo Clinic, Rochester, Minnesota; Mayo Clinic, Rochester, Minnesota; Department of Pharmacy, Mayo Clinic, Rochester, Minnesota; Mayo Clinic, Rochester, Minnesota; Mayo Clinic, Rochester, Minnesota; Department of Pharmacy, Mayo Clinic, Rochester, Minnesota; Mayo Clinic, Rochester, Rochester, Minnesota; Mayo Clinic, Rochester, Minnesota

## Abstract

**Background:**

Pharmacokinetics of beta-lactams vary widely in neonatal and pediatric patients due to developmental biology and critical illness. Cefepime (FEP), meropenem (MEM), and piperacillin/tazobactam (TZP) are highly susceptible to pharmacokinetic changes as hydrophilic, small molecules with renal clearance. Subtherapeutic levels are associated with clinical and microbiologic failure, and supratherapeutic levels associated with toxicity. Beta-lactam therapeutic drug monitoring (TDM) may optimize the effectiveness and safety of these agents. The purpose of this investigation is to describe the % of pediatric and neonatal patients with beta-lactam serum levels within target range.

**Methods:**

In August 2023, TDM launched at the Mayo Clinic Children’s Center for select patients treated with FEP, MEM, or TZP with expected durations ≥ 48 hours, or who were on kidney replacement therapy (KRT), extracorporeal membrane oxygenation (ECMO), weighed > 120 kg, had a BMI > 95^th^ percentile, or at the discretion of pediatric ID team. Validated drug assays were performed daily, Monday through Friday. A two-level sampling strategy was preferred with a peak 1 hour after the end of the infusion and a trough 30 minutes before the next dose. Empiric trough target ranges were set at FEP 8-50 mg/L, MEM 2-30 mg/L, and piperacillin 16-150 mg/L. These target ranges were adjusted with confirmed microbiology to a time above the minimum inhibitory concentration of the organism for 100% of the dosing interval (100%T > MIC).

**Results:**

From August 2023 to April 2024, 74 episodes of TDM were performed on 45 patients. Thirty-seven (82%) patients were in an intensive care unit (ICU) at the time of TDM, 8/45 (22%) of whom were in the neonatal ICU. Target range was achieved in 67% of patients on FEP, 50% on MEM, and 52% on TZP. Median (IQR) trough values were 22.1 (8.6, 44.2) mg/L for FEP, 0.6 (0, 4.1) mg/L for MEM, and 15.5 (3.8, 27.1) mg/L for TZP. Seven levels were above target for FEP and 0 for MEM and TZP. Seven levels were below target for FEP, 4 for MEM, and 11 for TZP.

**Conclusion:**

Implementation of a TDM program for FEP, MEM and TZP in pediatric and neonatal patients revealed drug levels frequently outside target range. TDM may facilitate dose individualization to optimize medication efficacy and minimize toxicity.

**Disclosures:**

**Raymond Stetson, MD, MS**, Moderna: Grant/Research Support **Christina G. Rivera (O'Connor), Pharm.D**, Gilead Sciences: Board Member **Erin F. Barreto, PharmD, MSc**, Baxter Health: Advisor/Consultant|Wolters Kluwer: Advisor/Consultant

